# Immobilization and 3D Hot-Junction Formation of Gold Nanoparticles on Two-Dimensional Silicate Nanoplatelets as Substrates for High-Efficiency Surface-Enhanced Raman Scattering Detection

**DOI:** 10.3390/nano9030324

**Published:** 2019-03-01

**Authors:** Yen-Chen Lee, Chih-Wei Chiu

**Affiliations:** Department of Materials Science and Engineering, National Taiwan University of Science and Technology, Taipei 10607, Taiwan; d10404013@mail.ntust.edu.tw

**Keywords:** gold nanoparticles, silicate platelets, Direct Blue dye, adenine, paraquat, surface-enhanced Raman scattering

## Abstract

We synthesize a high-efficiency substrate for surface-enhanced Raman scattering (SERS) measurements, which is composed of gold nanoparticles (AuNPs) on two-dimensional silicate nanoplatelets acting as an inorganic stabilizer, via the in-situ reduction of hydrogen tetrachloroaurate (III) by sodium citrate in an aqueous solution. Silicate platelets of ~1-nm thickness and various sizes, viz. laponite (50 nm), sodium montmorillonite (Na^+^–MMT, 100 nm), and mica (500 nm), are used to stabilize the AuNPs (Au@silicate), which are formed with uniform diameters ranging between 25 and 30 nm as confirmed by transmission electron microscopy (TEM). In particular, the laponite SERS substrate can be used in biological, environmental, and food safety applications to measure small molecules such as DNA (adenine molecule), dye (Direct Blue), and herbicide (paraquat) as it shows high detection sensitivity with a detection limit of 10^−9^ M for adenine detection. These highly sensitive SERS substrates, with their three-dimensional hot-junctions formed with AuNPs and two-dimensional silicate nanoplatelets, allow the highly efficient detection of organic molecules. Therefore, these Au@silicate nanohybrid substrates have great potential in biosensor technology because of their environmentally-friendly and simple fabrication process, high efficiency, and the possibility of rapid detection.

## 1. Introduction

Surface-enhanced Raman scattering (SERS) was first reported by Hendra, Fleischmann, and McQuillan [1], who studied the adsorption of pyridine onto roughened silver. The technique has since been widely used in biological [2], medical [3,4], environmental engineering [5], materials science [6,7], and quality analysis applications [8] because it offers increased Raman intensity by several orders of magnitude. Further, it offers sufficient sensitivity for single-molecule detection [9] and trace chemical analysis [10]. In this context, it is also known that rough metal nanoparticles (NPs) exhibit a large increase in surface plasmon excitation with electric field application, thus enhancing the Raman signal strength [11]. In particular, silver nanoparticles (AgNPs) exhibit the highest efficiency, and hence, they are widely used in SERS applications [12]. However, AgNPs can obstruct charge transfer during the assay of the molecule of interest, and they are also difficult to apply in bacteriology because of their sterilizing effect [13]. Instead, researchers have increasingly begun to use gold nanoparticles (AuNPs) for SERS substrates because they offer higher stability than AgNPs [14]. Furthermore, AuNPs possess low toxicity and are chemically inert, which translates to greater environmental safety.

A typical synthesis of substrates with high SERS activity involves growing AgNP nanoarrays on anodic aluminum oxide (AAO) [15]. It has been reported that the electromagnetic behaviors of AgNP arrays are virtually enhanced due to the spaces between the particle arrays because parts of the two- and three-dimensional (2D and 3D, respectively) hot-junctions formed are smaller than 10 nm. Fabrication methods of AuNPs include electron beam lithography [16], chemical vapor deposition [17], and the oxidation-reduction reaction [18]. Meanwhile, there is growing interest in the use of biodegradable and sustainable materials [19] for SERS. For Raman-scattering substrates, a major challenge lies in reducing or eliminating the generation of hazardous substances. In this regard, the method of low-temperature and facile sample preparation in aqueous solutions represents an environmentally-friendly and user-friendly approach [20,21]. In the oxidation-reduction approach, various reducing and protecting agents have been reported to achieve the eco-friendly synthesis of AuNPs for SERS-active substrates. Reported green reducing agents include tea polyphenols [22], hyaluronic acid [23], and ascorbic acid [24]. Among the many agents available, sodium citrate is the most commonly applied as both dispersing agent and reducing agent to stabilize the NPs. On the other hand, layered silicate clays in organic polymers acting as composites have been intensively studied as substrate materials [25,26]. Meanwhile, to ensure that the NPs remain highly homogeneous, dispersants are commonly required for interacting with the particle surface [27,28,29,30,31]. The use of one or more kinds of layered clay can create highly stable metal NPs for use in oxidation–reduction reactions [32]. Due to their advantageous chemical properties, it has been posited that such clays can form effective adsorbents for stabilizing heavy-metal NPs [33,34,35]. Recently, 3D hot-junctions of AgNP/2D nanomaterial hybrids have been used as SERS substrates for various biochemical analyses [36,37,38,39].

Against this backdrop, here we report a new environmentally-friendly substrate composed of Au@silicate nanoplatelets for use in high-sensitivity SERS measurements. AuNPs with a narrow size distribution were finely dispersed in a nanohybrid surfactant, and clay particles with different sizes were tested as dispersants. The sensitivity of the fabricated SERS substrate was tested for adenine [40], Direct Blue 200 [41], and paraquat [42], which are important target chemicals in medical science and environmental engineering. Au@laponite was found to afford the best SERS substrate with great potential for application to biosensing technology.

## 2. Materials and Methods

### 2.1. Materials

Hydrogen tetrachloroaurate (III) (HAuCl_4_, >99.5%) and trisodium citrate dehydrate (C_6_H_5_Na_3_O_7_, 100%) were obtained from Alfa Aesar Chemical (Ward Hill, MA, USA) and Showa Chemical Industry Co. (Tokyo, Japan), respectively. Laponite, an artificial clay with the composition of 26.7 wt% Si, 15.3 wt% Mg, 0.1 wt% Al, 2.3 wt% Na, and 3.3 wt% Fe, was obtained from Elgin Corporation Taipei, Taiwan). Further, Na^+^–montmorillonite (MMT) was obtained from Nanocor Inc. (Minerals Technologies Inc.,New York, USA, 31.81 wt% Si, 9.37 wt% Al, 4.0 wt% Na, and 6.6 wt% Fe). Synthetic fluorinated mica (ME-100), which is a layered silicate clay with 24.9 wt% Si, 12.12 wt% Mg, 0.4 wt% Al, 5.8 wt% Fe, and 2.6 wt% Na, was obtained from CO-OP Chemical Co., Tokyo, Japan. The compounds used for detection were adenine (powder, 99.9%, Sigma-Aldrich, St. Louis, MO, USA), Direct Blue 200 (Nippon Kayaku Co., Tokyo, Japan, supplied by Chung Fu Dyestuffs Co., Taoyuan, Taiwan), and paraquat (Echo Chemical Co., Miaoli, Taiwan). All the utilized glassware was cleaned with deionized water, rinsed with acetone, and stocked at 105 °C before use.

### 2.2. Preparation of Au@silicate Hybrid Suspensions

The AuNPs stabilized on the layered clay structure were prepared as follows. Laponite, MMT, or mica powder was well dispersed in water (5 mL, 5 wt%), with the solution being stirred at 80 °C for 1 h. Next, HAuCl_4_ (5 mL, 6 mM) was added to the clay suspension with constant stirring to allow the intercalation of Au^3+^ ions. This mixture was injected into a solution of sodium citrate (10 mL, 0.02 M) and maintained at 60 °C with constant stirring by use of a magnetic agitator. After 6 h of redox reaction, the color of the aqueous solution changed from light yellow to pink. The presence of the Au NPs was confirmed by UV–Vis absorption spectroscopy, with the appearance of the typical absorption peak at ~522 nm indicating the reduction of Au^3+^ to Au^0^. After centrifugal concentration, an AuNP suspension of 1 mg/mL was obtained, which was characterized using a UV–Vis spectroscopy (Kyoto, Japan), transmission electron microscopy (TEM, Tokyo, Japan) system, a zeta-potential analyzer (New Taipei, Taiwan), and a LUMiSizer dispersion analyzer (Berlin, Germany).

### 2.3. Preparation of SERS Samples

SERS substrates were prepared by drop-coating the Au@silicate solution on a clean glass substrate of dimensions 5 mm × 5 mm and then heating in an oven at 60 °C to form a thin film. For SERS characterization, the Au@silicate hybrid substrate was soaked perpendicularly in 1 mL analyte solution for 60 s to achieve surface adsorption, and subsequently, the water was dried by evaporation. The analyte solutions were prepared by dissolving adenine, Direct Blue 200, or paraquat at concentrations of 10^−3^, 10^−4^, 10^−5^, 10^−6^, 10^−7^, 10^−8^, and 10^−9^ M. Finally, the prepared substrates were subjected to Raman spectromicroscopy measurements at four different points on each SERS sample surface, and high reproducibility was observed.

### 2.4. Characterization and Instruments

Field-emission scanning electron microscopy (FE-SEM) images were obtained by means of a Zeiss EM 902A system with the use of a Pt sputter coating target and a field-emission gun operated at 15 kV. The optical properties of the AuNP suspensions were examined via UV–Vis spectroscopy (Shimadzu UV-2450, Kyoto, Japan) over 400–800 nm with a scan rate of 400 nm/min. Further, TEM was performed with the use of a Zeiss EM 902A system operated at 80 kV. The zeta potential of the solution was measured by means of a Zetasizer Nano-Zs90 instrument. The stability of the AuNP suspension was characterized with a LUMiSizer-6111 system using a laser wavelength of 865 nm and a 2048-pixel charge-coupled device (CCD) sensor scanning once per minute for a total of 5 h at 35 °C. Raman spectra were recorded and integrated with a HORIBA iHR550 Raman microscope system (Protrustech Corp., Ltd., Tainan, Taiwan), and a silicon CCD camera was used for light detection. A laser operating at λ = 532 nm was focused via a 50× objective lens (Olympus BX-41) onto the sample with an excitation area of ~4 μm^2^. The SERS spectra were acquired over 1 s. For each analyte, SERS spectra were collected at an average of 10 randomly selected positions on the AuNP substrate.

## 3. Results and Discussion

### 3.1. Dispersion Mechanism for the Au@silicate Nanohybrids

**Figure 1:**
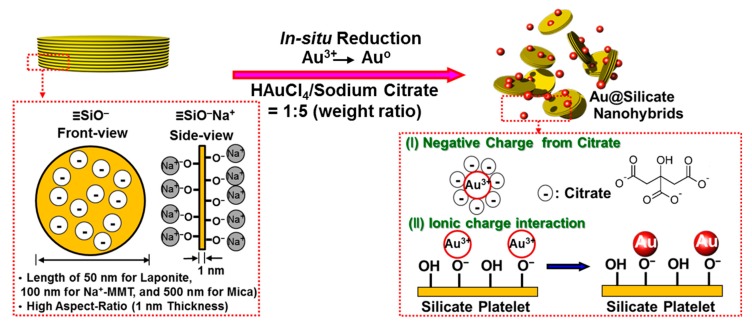
Schematic of dispersion mechanism of Au@silicate hybrids due to non-covalent interactions: (I) negative charges on the particles from adsorbed citrate anions and (II) ionic interactions between Au nanoparticles (AuNPs) and silicate platelets that can prevent further particle aggregation. MMT is montmorillonite.

The dispersion mechanism of Au@silicate nanohybrids is depicted schematically in Figure 1. On the flat surface of silicate clay nanoplatelets, the reduction of HAuCl_4_ in solution affords nanometer-scale gold particles. The layered silicate clays of laponite, Na^+^–MMT, and mica have different platelet dimensions of 50 nm × 50 nm × 1 nm, 100 nm × 100 nm × 1 nm, and 500 nm × 500 nm × 1 nm, respectively [26]. These silicate nanoplatelets can disperse rapidly in water without the need for a high shear force, and the colorless dispersion does not hinder laser penetration in Raman measurements. These inorganic materials are also non-toxic, non-flammable, and stable at high temperatures. In general, silicate clays have a cationic lamellar structure. The cations can exchange easily in aqueous solutions, and the strong negative charge on the layers permits ready adsorption of proteins, other organic macromolecules, and viruses onto the surface. The cationic exchange reaction of clays is very fast, particularly for polyvalent cations. The cation exchange capacity (CEC) of laponite is 75 milliequivalents (meq) per 100 g, while those of MMT and mica range up to 120 meq/100 g [43]. The –OH groups on the base plane of the clay and the sheet of Si–O tetrahedra allow the d-spacing to change under external physical forces or thermal effects. The highly charged Au ions replace monovalent Na^+^ with little pH dependence. During reduction of the intercalated Au ions into AuNPs, the clay does not react but forms a coating on the NPs. Due to its surface charge in solution, the coated clay stabilizes the NPs by electrostatic attraction.

On the other hand, sodium citrate serves as both a reductant of gold ions on the base plane of clay as well as a stabilizer of the formed AuNP colloid, because the citrate anion adsorbed onto the NP surface generates a negatively charged layer to suppress particle collision due to Brownian motion. The hydroxyl group of sodium citrate is effective for reducing Au^3+^, and the remaining sodium citrate protects the NPs to achieve uniform dispersion (whereas the dispersion easily becomes aggregated with other salts). We compared a series of Au@silicate composite solutions prepared by reducing HAuCl_4_ in laponite, montmorillonite, or mica after swelling of these hydrophilic clays in water. The initial HAuCl_4_ aqueous solution after injection into sodium citrate appeared golden yellow, and the color gradually changed to pink and red as the redox reaction proceeded. Here, we note that the color of the mixed aqueous solution depends on the size of AuNPs to achieve resonance with the visible light frequency, which is known as the surface plasmon resonance phenomenon.

### 3.2. AuNP Stabilization by Two-Dimensional Silicate Nanoplatelets at Various Weight Ratios

**Figure 2:**
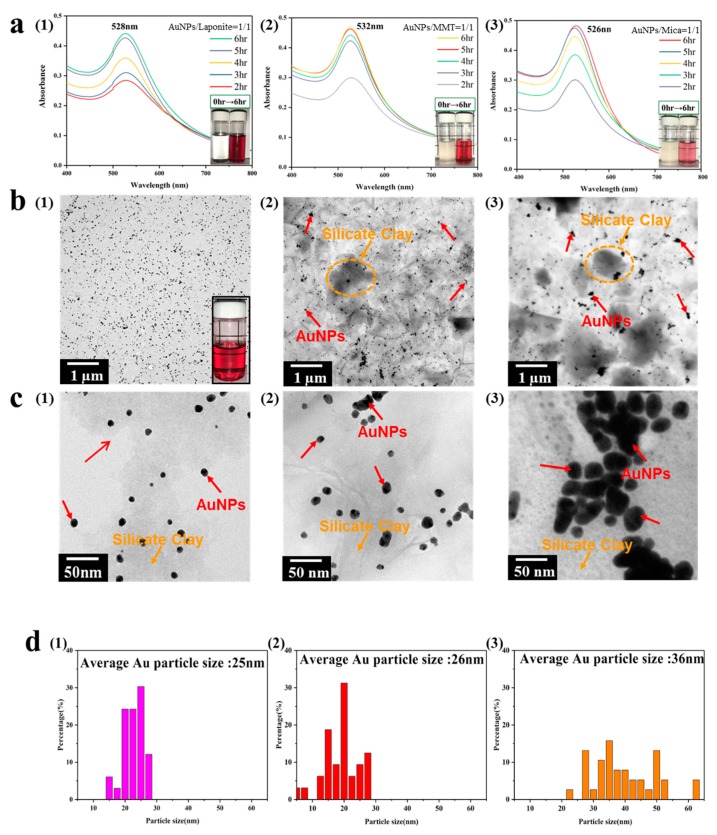
(**a**) UV–Vis absorption spectra of (1) Au@laponite, (2) Au@MMT, and (3) Au@mica at different reaction times and a constant weight ratio of 1:1. (**b**,**c**) Transmission electron microscopy (TEM) micrographs at different magnifications. The inset in **b** (1) shows a strong dispersion after 3 months of undisturbed hybrid suspension. (**d**) Particle size histograms of AuNP suspensions based on TEM images.

The zeta potentials, UV absorption spectra, and average Au particle size of the composite solution, after reduction of Au^3+^ to Au^0^, are listed in Table 1. Successful preparation of AuNPs via reduction by sodium citrate was confirmed by UV–Vis absorbance spectroscopy (Supplementary Material Figure S1), and the resulting AuNPs were ~20 nm in size and of different shapes. For a 1:1 weight ratio between HAuCl_4_ and laponite, the solution appeared scarlet in color. The UV–Vis absorption spectra acquired at different redox times during the reaction are shown in Figure 2a. The absorption peak at 528 nm was attributed to the free conduction electrons photoexcited on the surface of particles. The intensity of the surface plasmon resonance (SPR) band increased upon increasing the redox time from 30 min to 7 h, and it stopped increasing when the reduction reaction was complete. When the number of AuNPs increases, the absorbance also increases slightly and is red-shifted, because a higher metal concentration is expected to generate SPR. In the study, upon increasing the HAuCl_4_/laponite ratio from 1:1 to 1:20, we observed that the color of the solution changed to purplish red and detected the SPR absorption band at ~533 nm. In addition, the size of AuNPs (~35 nm in this case) immobilized on the surface depends on the presence of silicate nanoplatelets. Our TEM observations indicated that the AuNPs were coated with 3 nm-thick layers of laponite, as shown in Figure 2b. The Brownian motion of AuNPs, which is the non-stop random motion of particles suspended in solution, could be suppressed because of the clay platelets interacting via van der Waals forces. Figure 2c shows the layered structure with high laponite content, and the image confirms that the AuNPs can enter between the layers for better stabilization and that the surface of laponite is covered with Au particles.

For the other two clays, Na^+^–MMT and mica, the AuNPs exhibited a wide size distribution of 30–50 nm (Figure 2d and Table 1). Similar to laponite, MMT is a typical bentonite, a natural mineral with high CEC. For a HAuCl_4_/MMT weight ratio of 1:1, the color of the solution appeared wine red, thus indicating the ability of MMT to stabilize the AuNPs. For higher MMT content, the color became deeper (close to purple), although the maximum absorbance wavelength remained at ~530 nm. From the TEM image, we noted that the average particle size did not change significantly with the MMT content, but the size distribution was broad, and a layered structure of MMT could be observed. A brown precipitate appeared in the solution for HAuCl_4_/MMT = 1:20, and the CEC of MMT was estimated as 0.21. Consequently, the number of Au ions was less than the total CEC of MMT, and the excess MMT was precipitated instead of stabilizing the AuNPs.

When compared with the other two silicates, the mica platelets were relatively large (>500 nm × 500 nm). Without an external force to “swell” them, they precipitated at the bottom and did not stabilize the AuNPs very well. We observed that when the HAuCl_4_/mica ratio ranged from 1:1 to 1:20, the SPR absorption peaks at 526–527 nm all appeared similar, without showing any redshift. The likely explanation is that mica did not participate in the reaction producing the AuNPs. These differences originated from the bond strength in the charged layer in the basal plane of the clay, and the clay layers were bonded together by van der Waals forces. The clay layer was negatively charged, and the exchanged metal ions were bonded by electrostatic attraction. Smectites such as laponite and MMT have relatively weaker bonds; therefore, polar solvent molecules such as water can enter their base plane to swell them, subsequently leading to the exchange of metal ions in water. On the other hand, mica has higher charge on its layers, and the resultant strong ionic bond does not allow interlayer expansion. Consequently, the tetrachloroaurate ions cannot enter the interlayer, but only undergo reduction on the edge of mica without any stabilization effect. Upon comparing different silicate nanoplatelets, we concluded that the surface area with –OH groups on the base plane of clay is the controlling factor in the production of AuNPs.

The stability of the composite solution was also assessed by means of a LUMiSizer system. The simulation results of the NP movement over a long time period are presented in Supplementary Material Figure S2. The LUMiSizer system can be used to measure the positions of particles in an aqueous solution based on the intensity of transmitted light under high-speed rotation. Here, we note that centrifugation saves test time relative to sedimentation under gravity, and the use of a surface light source aids in avoiding the time difference due to traditional scanning. The light penetration strength, as calculated by the Lambert–Beer law, is the intensity difference between the incident and transmitted light signals over an identical light path length. The first recorded transmission profile of the Au@silicate composite solution determined the overall intensity profile (Supplementary Material Figure S2a–c). Upon centrifugation and increase in the reaction time, AuNP sedimentation proceeded from left to right, thereby increasing the transmittance of the solution. The red lines denote the first profile, whereas the final ones are depicted in green to show the moving liquid–solid boundary with increasing transmission. Laponite effectively clarified the suspension over time, which led to an increase in transmission from 23% to 65%. In addition, the separation behaviors of the individual samples were compared, as seen in Figure S2d. The spread rates of the three complex solutions were calculated by means of Stokes’ law to be 3.16 (laponite), 5.24 (MMT), and 6.70 mm/min (mica). Au@laponite had the slowest spread rate among the three, thereby indicating that laponite had the strongest stabilizing effect on the AuNPs.

**Figure 3:**
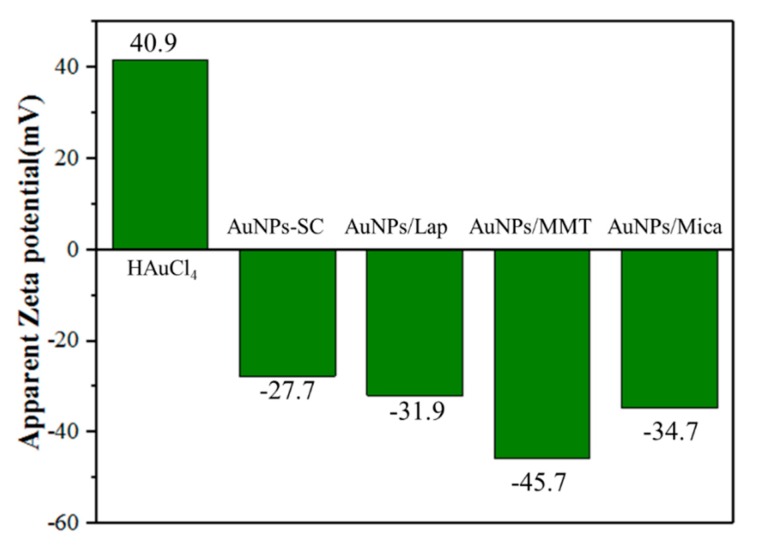
Zeta potential of Au@silicate platelets reduced by NaBH_4_ and sodium citrate. For sodium citrate, the weight ratio of AuNP:silicate clay (SC) was 1:1. Lap is laponite.

The zeta potential was used to analyze adsorption onto the clay and AuNPs, and these results are presented in Figure 3 and Table 1. When HAuCl_4_ was dissolved in deionized water, the zeta potential of the resultant Au^3+^ solution was 41.7 mV. This value became −33.4 mV with the use of sodium citrate as the reductant without adding any dispersant, because the citrate created a stabilizing coating on the AuNPs. Clays swollen in water exhibited zeta potentials between −44 and −51 mV. After the clay underwent cation exchange in the HAuCl_4_/clay system with weight ratios from 1:1 to 1:10, the AuNPs intercalated into the clay, and the zeta potential improved and approached the interface potential balance. With an increase in the weight ratio of clay, the exchange effect, and thus the zeta potential, became saturated.

### 3.3. Raman Shift of SERS Samples in Au@silicate Nanohybrids

**Figure 4:**
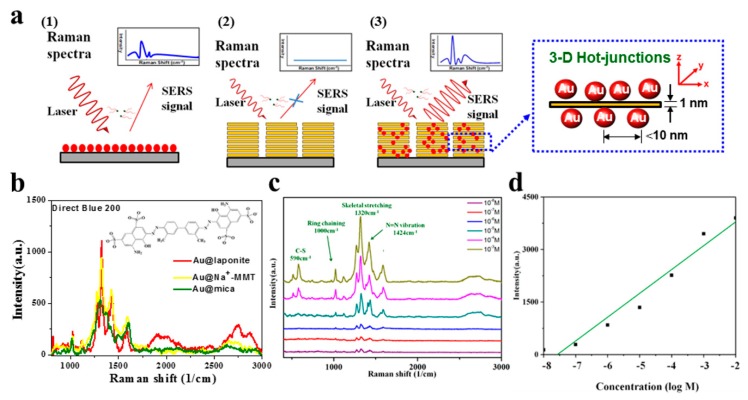
(**a**) Schematic of Au@silicate surface–enhanced Raman scattering (SERS) substrate interacting with organic analyte molecules. (1) AuNPs–SC, (2) pristine laponite, and (3) Au@laponite. (**b**) SERS intensities of 10^−5^ M Direct Blue 200 solution on AuNP substrates stabilized by different nanosilicates (weight ratio of 1:1). (**c**) SERS spectra of Direct Blue adsorbed onto Au@laponite films with a precursor weight ratio of 1:1, and (**d**) calibration curve for the SERS spectra at 1320 cm^−1^.

The AuNP aqueous solution was drop-coated onto glass for use as high-efficiency SERS substrates, as schematically illustrated in Figure 4a, and the Raman spectra are shown in Supplementary Material Figures S3 and S4. The SERS efficiency was evaluated using Direct Blue 200 as the standard Raman-active probe molecule (Figure 4b). Direct Blue is a family of water-soluble dyes commonly used in industry, and they are generally seen in wastewater from dyeing and finishing plants. In this study, we measured a series of SERS spectra of Direct Blue adsorbed onto Au@silicate (prepared at the weight ratio of 1:1) as hybrid substrates to characterize the substrate sensitivity. Furthermore, a video showing rapid molecular detection by 3D hot-junctions of the Raman-enhancing silicate platelet nanohybrid is provided in Supplementary Material Video S1. The enhancement for AuNPs stabilized by different clays is shown in Figure 4b. Direct Blue 200 in water easily hydrated and contacted the clay. Au@laponite exhibited the highest intensity because it had good optical transparency and narrow particle size (the SERS enhancement depends on the surface of the AuNPs). Au@MMT also demonstrated good intensity for Direct Blue, but the results were less reproducible because of the uneven NP size. Au@mica exhibited low intensity and larger AuNPs owing to the 3D hot-junction behavior with AuNPs adsorbed between the clay nanosheets, similar to the cases of laponite and MMT, to allow the detection of probe molecules. In the study, AuNPs prepared without adding silicate were also tested for comparison.

The substrate of Au@laponite was applied to measure Direct Blue 200, and the sensitivity was characterized by the detection limit. The main Raman bands of Direct Blue 200 in water are located at 513, 590, 1025, 1120, 1280, 1320, 1425, and 1580 cm^−1^ (Figure 4c). The peaks corresponding to the N=N double bond (1425 cm^−1^) and the ring breathing mode (1320 cm^−1^) are more intense than other peaks, such as those corresponding to the vibrations of C–N (1580 cm^−1^) and C–S aliphatic bonds (500–600 cm^−1^) [40]. The AuNP substrates exhibited high sensitivity for the Direct Blue solution, with a detection limit of 10^−8^ M. For quantitative analysis, the strongest signal of Direct Blue 200 (1320 cm^−1^) was used to create a calibration curve (Figure 4d). Linear regression indicated good linearity with high coefficient of determination (R^2^ = 0.9586).

**Figure 5:**
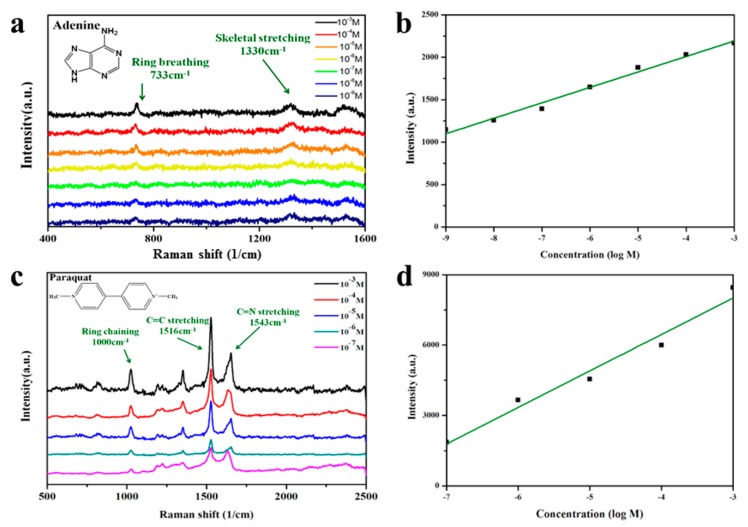
(**a**) Surface-enhanced Raman scattering (SERS) spectra of adenine adsorbed onto Au@laponite hybrid films with precursor weight ratio of 1:1 and (**b**) calibration curve for the SERS spectra at 733 cm^−1^. (**c**) SERS spectra of paraquat adsorbed on the Au@laponite hybrid films with precursor weight ratio of 1:1 and (**d**) calibration curve for the SERS spectra at 1516 cm^−1^.

We also tested adenine and paraquat as Raman-active probe molecules. Adenine is a component of DNA and plays a variety of roles in biochemistry. The peak of adenine originates from the Raman-active bending and stretching modes of the benzene ring. The peaks at 733 and 1331 cm^−1^ can be assigned to the C–H breathing and N–C vibrational modes, respectively, of the adenine ring [41]. Other bands at 680, 1040, 1160, 1240, 1330, 1380, and 1440 cm^−1^ become drastically weaker in dilute solutions. In our study, the detection sensitivity of SERS for adenine was very high and the detection limit was 1 × 10^−9^ M (Figure 5a,b). The regression curve for the intensity of the 733-cm^−1^ peak indicated R^2^ > 0.98, which means that the substrate exhibited a highly reproducible performance, and the 3D hot-junctions were distributed evenly to achieve high efficiency.

The organic compound paraquat is one of the most widely used herbicides. It is highly toxic to human beings and animals and has thus been banned or restricted in many countries. For paraquat, the detection limit of the Au@laponite films with a precursor weight ratio of 1:1 was below 1 × 10^−7^ M. Characteristic peaks of paraquat (Figure 5c,d) are located at 1000, 1516, and 1624 cm^−1^, corresponding to ring vibration, C=C stretching, and C=C stretching, respectively [42]. For quantitative analysis, a regression curve was plotted to show that the accuracy can reach 95%, thereby indicating potential application for water quality measurement. We measured a series of SERS spectra of the three probe molecules after adsorption onto Au@laponite hybrid substrates to characterize the sensitivity of the proposed SERS substrate. Although the spectra contained some noise, the target molecules could still be detected from the solution even at trace quantities. Thus, the three molecules that are important in medicine, food, and environmental analyses can be detected by SERS with high sensitivity by means of our new AuNP-based active substrate. Supplementary Material Table S1 compares the SERS substrates based on various Au-related nanomaterials in terms of the “green fabrication” method, detected concentrations, cells detected or molecular analysis, excitation sources, enhancement factor, and limit of concentration.

## 4. Conclusions

We developed a series of nanohybrids for AuNPs synthesized by sodium citrate reduction and dispersed with clay nanoplatelets for use as sensitive substrates for surface-enhanced Raman scattering (SERS) measurements. Coating the AuNPs on a layer of laponite in solution afforded electrostatic attraction from their surface charges. Our TEM analysis confirmed the successful preparation of AuNPs with a narrow size distribution of 25 nm. As a SERS substrate, the composites of spherical AuNPs and clay afford high efficiencies for measuring Direct Blue, adenine, and paraquat with low detection limits of 10^−8^ M, 10^−9^ M, and 10^−7^ M, respectively. We note here that the SERS substrate allowed the detection of an industrial pollutant, a model target biomolecule, and a herbicide residue. These efficient low-cost SERS substrates, prepared using a green fabrication process, have potential application in environment inspection, biosensor technology, and food safety monitoring.

## Figures and Tables

**Table 1:** Reduction of AuNPs with different amounts of clay hybrids.

HAuCl_4_/Silicate Platelets (Weight Ratio) ^a^	Au^3+^/CEC (Molar Ratio) ^b^	Solution Color	Zeta Potential (mV)	UV–Vis Absorption (nm)	Average Ag Particle Size by TEM (nm) ^c^
**AuNPs**					
1/0	0	Pink	–27.7	523	25 ± 3
**Au@laponite**					
					
1/1	6.77	Scarlet	–31.9	528	27 ± 5
1/5	1.35	Cerise	–49.4	526	28 ± 7
1/10	0.68	Purplish red	–52.8	533	26 ± 5
1/20	0.34	Purplish red	–56	528	28 ± 5
5/1	33.84	Scarlet	–40.8	528	27 ± 7
10/1	67.69	Scarlet	–34.3	530	26 ± 12
**Au@Na^+^–MMT**					
1/1	4.23	Wine red	–45.7	532	32 ± 7
1/5	0.85	Wine red	–46.3	529	29 ± 6
1/10	0.42	Purplish red	–46.0	530	37 ± 8
1/20	0.21	Purple with precipitate	–48.2	533	34 ± 7
5/1	21.15	Red	–40.1	523	35 ± 11
**Au@Mica**					
1/1	4.23	Pink with precipitate	–34.7	526	50 ± 8
1/5	0.85	Pink with precipitate	–42.3	523	-
1/10	0.42	Pink with precipitate	–39.3	526	-
1/20	0.21	Pink with precipitate	–34.7	527	-
5/1	21.15	Scarlet	–33.4	527	32 ± 7

^a^ The weight ratio is calculated on the basis of HAuCl_4_/silicate and ranges from 1:0 to 10:1. ^b^ Molar ratio of Au^3+^/cation exchange capacity (CEC) of silicate platelets. ^c^ Average particle sizes of gold were measured by TEM.

## Supplementary Materials

The Supplementary Materials are available online at https://www.mdpi.com/2079-4991/9/3/324/s1. Figure S1: The UV-visible absorption sprctra of AuNPs reduced by reduced by sodium citrate. Figure S2: The movement and the separation behaviors of AuNPs@silicate under the LUMisizer. Figure S3 and S4: The Raman spectra of AuNPs, AuNPs@silicate and silicate can illustrate the 3-D hot junction. Video S1: The Raman detection of the AuNPs@silicate. Table S1: The Au-related nanomaterials in terms of the “green fabrication” method used as the SERS substrates.

## Abbreviations

Surface-enhanced Raman scattering (SERS), nanoparticle (NP), anodic aluminum oxide (AAO), montmorillonite (MMT), field emission scanning electron microscopy (FE-SEM), transmission electron microscopy (TEM), charge coupled device (CCD), cation exchange capacity (CEC), surface plasmon resonance (SPR).
