# Supplementary material for: Immobilization and 3D Hot-Junction Formation of Gold Nanoparticles on Two-Dimensional Silicate Nanoplatelets as Substrates for High-Efficiency Surface-Enhanced Raman Scattering Detection
Source: Nanomaterials (Basel). 2019 Mar 1;9(3):324. doi: 10.3390/nano9030324 (PMC6473534; doi:10.3390/nano9030324)
Supplement: Supplementary file 1 [file nanomaterials-09-00324-s001.zip › nanomaterials-435087-SI-for final/nanomaterials-435087-SI-for final.pdf]

Supporting Information

Immobilization and 3D Hot-Junction Formation of Gold Nanoparticles on Two-Dimensional Silicate Nanoplatelets as Substrates for High-Efficiency Surface-Enhanced Raman Scattering Detection

Yen-Chen Lee and Chih-Wei Chiu*

Department of Materials Science and Engineering, National Taiwan University of Science and Technology, Taipei 10607, Taiwan

* Corresponding author:

Tel: +886-2-2737-6521; Fax: +886-2-2737-6544; E-mail: cwchiu@mail.ntust.edu.tw (C.-W. Chiu).

Contents:

FIGURE CAPTIONS

Fig. S1. (a) UV-visible absorption spectra of AuNPs reduced by sodium citrate. (b and c) TEM micrographs at different magnifications.

Fig. S2. Light transmission through the AuNP composite solution with Au:silicate nanoplates in 1:1 weight ratio under high-speed rotation. The silicate nanoplatelets were (a) laponite, (b) montmorillonite, and (c) mica. (d) Spread rate of the three Au@silicate complex solutions.

Fig. S3. SERS intensity of 10^{-5} M direct blue 200 on different substrates: AuNPs, nanohybrids, and AuNPs stabilized with laponite silicate platelets in 1:1 weight ratio.

Fig. S4. SERS intensity of Au@laponite hybrid substrates.

TABLE LIST

Table S1. Summary of various Au-related nanohybrid substrates for SERS.

VIDEO

Video S1. A movie demonstration of rapid molecular detection by 3D hot-junctions of Raman enhancing AuNP@silicate platelet nanohybrid substrate.

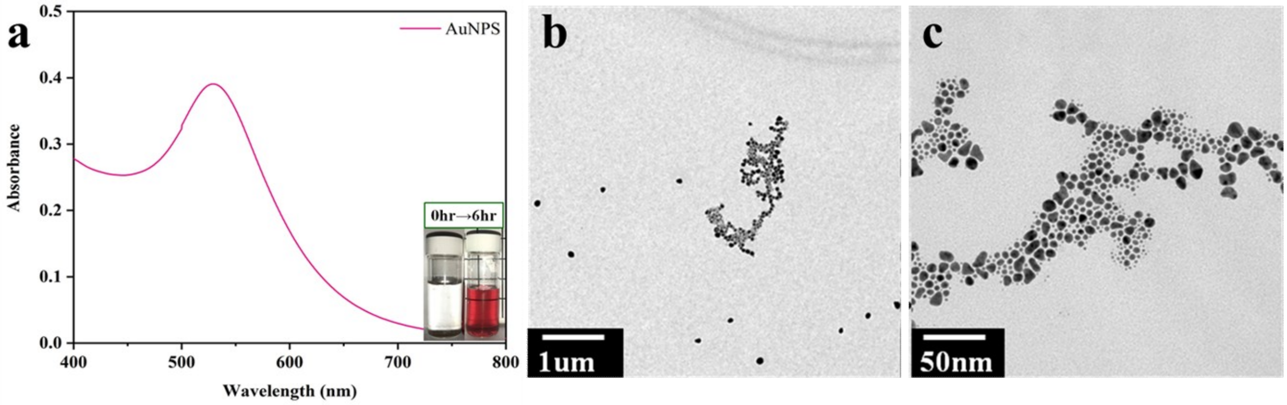

Fig. S1. (a) UV-visible absorption spectra of AuNPs reduced by sodium citrate. (b and c) TEM micrographs at different magnifications.

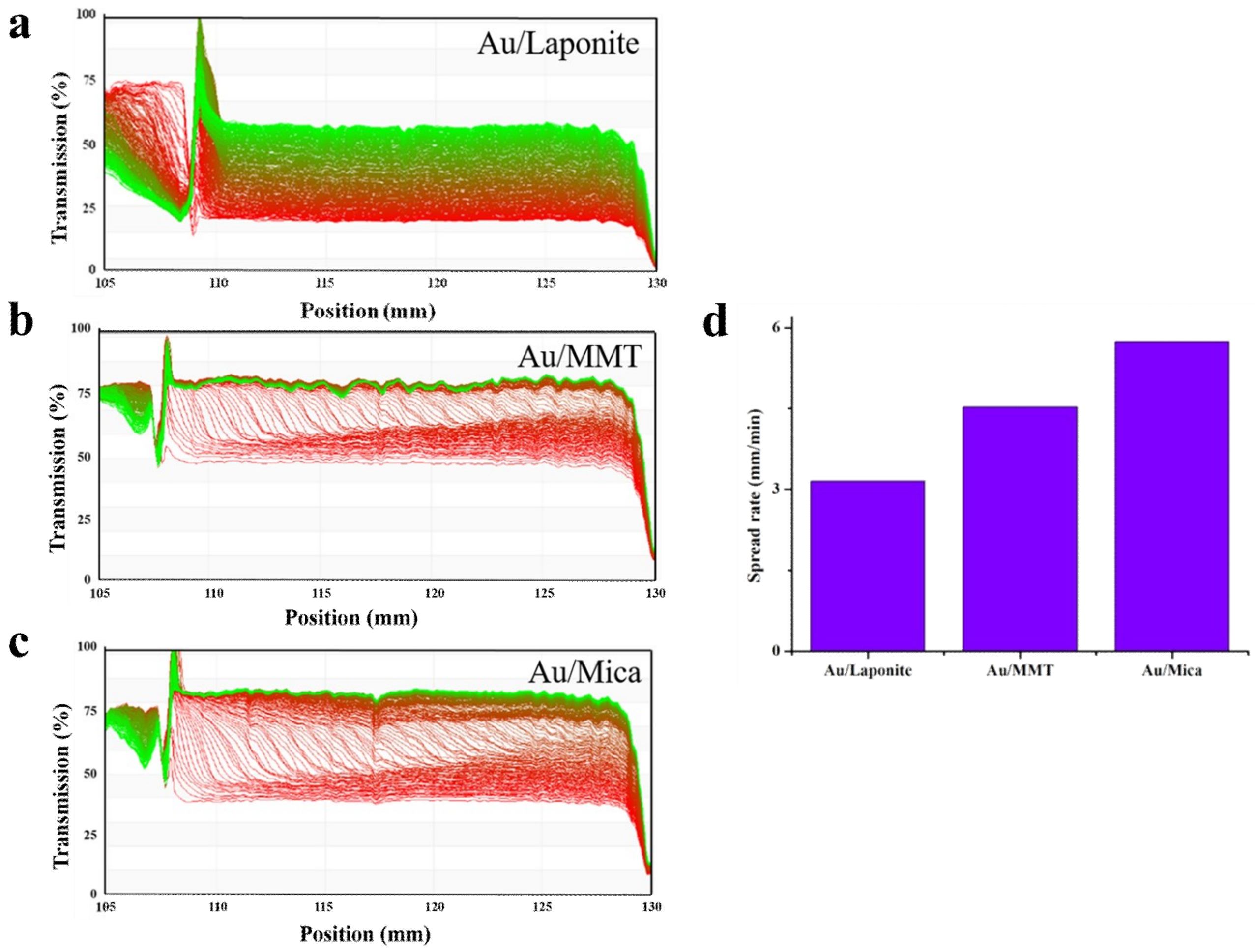

Fig. S2. Light transmission through the AuNP composite solution with Au:silicate nanoplates in 1:1 weight ratio under high-speed rotation. The silicate nanoplatelets were (a) laponite, (b) montmorillonite, and (c) mica. (d) Spread rate of the three Au@silicate complex solutions.

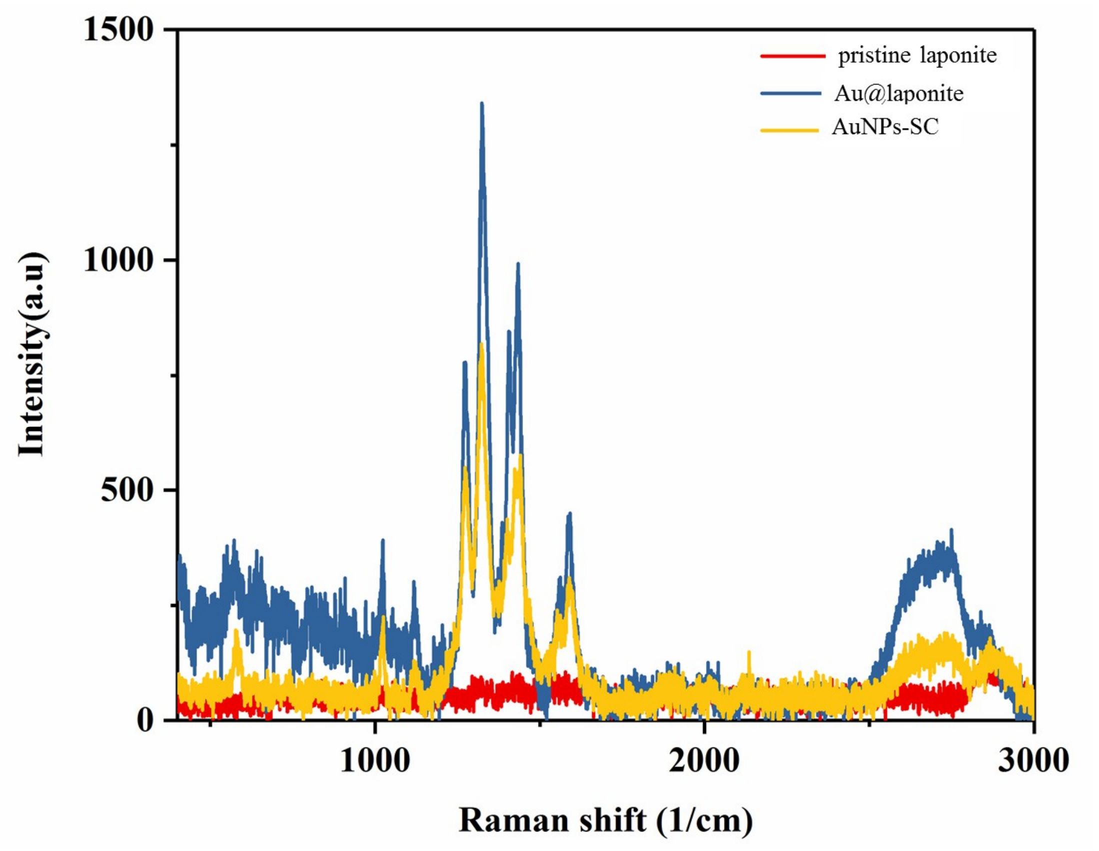

Fig. S3. SERS intensity of 10^{-5} M direct blue 200 on different substrates: AuNPs-SC, pristine laponite, and AuNPs stabilized with laponite silicate platelets in 1:1 weight ratio.

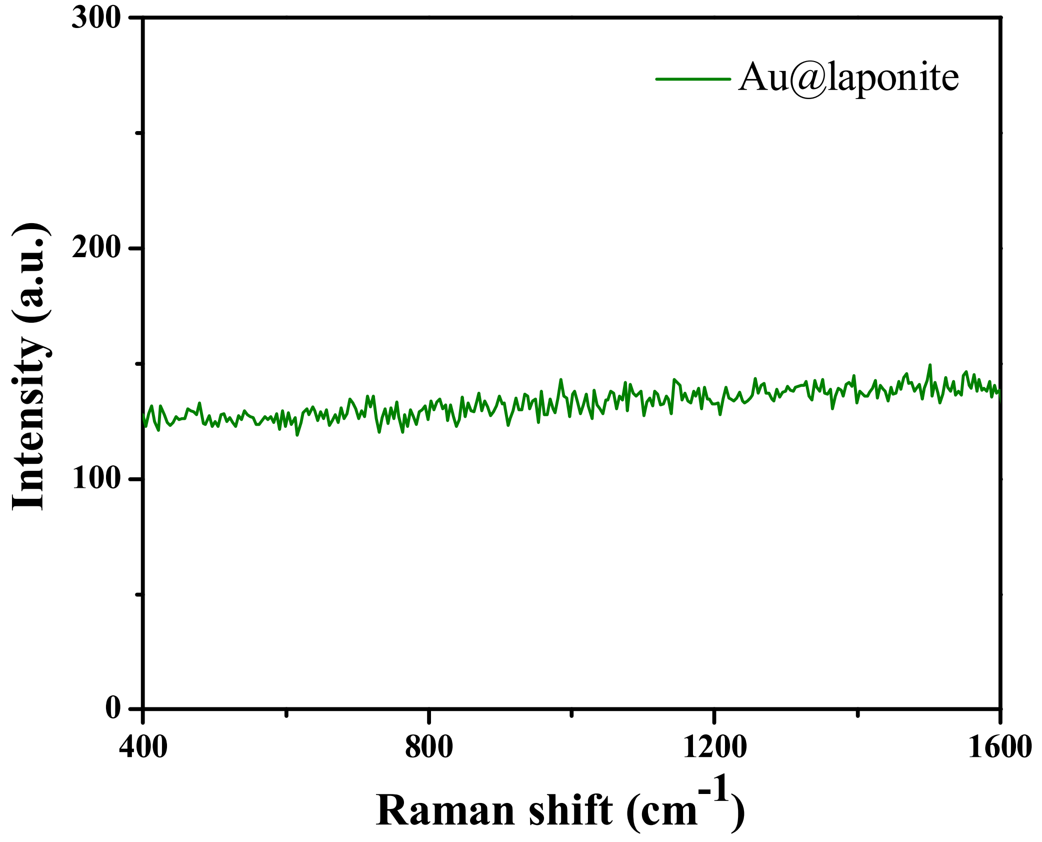

Fig. S4. SERS intensity of Au@laponite hybrid substrates.

Table S1. Summary of various Au-related nanohybrid substrates for SERS.

Hybrid composition	Reducing agent	Analyte molecule or cells detected	Laser excitation wavelength (nm)/source power (mW)	SERS LOE[1]/EF Value[2]	Reference
Silver	Hyaluronic Acid (HA)	Hyaluronic Acid (HA)	785/17	Not given	S1
AuNPs	Sodium citrate	4-Mercaptopyridine (4-Mpy)	633/17	10^{-9}M/1.3×10^{5}	S2
AuNTs	$NaBH_4$	Thiols Benzenethiol (BT)	785/5	10^{-8}M/1.2×10^{5}	S3
Au-Ag Alloy Nanourchins	l-Dihydroxphenylalanine (L-Dopa)	Crystal violet (CV)	633/Not given	10^{-14}M/1.6×10^{9}	S4
Gold nanocrystals (AuNCs)	Dimethyl formamide (DMF)	4-Mercaptobenzoic acid (MBA)	785/Not given	Not given/ 4.5×10^{8}	S5
Au nanstar@SiO_2	Trisodium citrate	Glucose	785/Not given	1.6×10^{-5} M/Not given	S6
Fe_2Ni@Au	Hydrazine hydrate	Doxorubicin (DOX)	633/8	8 mug mL^{-1}/Not given	S7
AuNPs@MoS_2	Intercalation-exfoliation method	Rhodamine 6G (R6G)	633/20	5×10^{-7}M/8.2×10^{5}	S8
Fe_3O_4@Au	4-Mercaptobenzoic acid (MBA)	4-Mercaptobenzoic acid (MBA)	632.8/5	100 fg mL^{-1}/Not given	S9
AuNPs	Sodium citrate	Methylene blue (MB)	633/2	2 ng mL^{-1}/2.6×10^{4}	S10
AuNPs grown on Ge wafer	Ge	Rhodamine 6G (R6G)	633/Not given	10^{-7}M/4.5×10^{6}	S11
Fe_3O_4/Au cluster/shell	Sodium citrate	Prostate Specific Antigen (PSA)	785/100	0.75 ng mL^{-1}/9.27×10^{4}	S12
AuNPs/Silicate platelets	Sodium citrate	(1) Direct Blue 200<br>(2) adenine<br>(3) paraquat	532/20	(1) 10^{-8}M<br>(2) 10^{-9}M<br>(3) 10^{-7}M	Our work

[1] LOE: limit of concentration. [2] EF: Enhancement factor.

References

[S1] N. Xia, Y. Cai, T. Jiang. Green synthesis of silver nanoparticles by chemical reduction with hyaluronan, Carbohydr. Polym. 86 (2011) 956–961.

[S2] J. Chen, Y. Huang, P. Kannan, L. Zhang, Z. Lin, J. Zhang, T. Chen, L. Guo. Flexible and adhesive surface enhance Raman scattering active tape for rapid detection of pesticide residues in fruits and vegetables, Anal. Chem. 88 (2016) 2149–2155

[S3] L. Scarabelli, M. Coronado-Puchau, J.J. Giner-Casares, J. Langer, L.M. Liz-Marzán. Monodisperse gold nanotriangles: size control, large-scale self-assembly, and performance in surface-enhanced Raman scattering, ACS Nano 8 (2014) 5833–5842.

[S4] Z. Liu, Z. Yang, B. Peng, C. Cao, C. Zhang , H. You, Q. Xiong, Z. Li, J. Fang. Highly sensitive, uniform, and reproducible surface-enhanced Raman spectroscopy from hollow Au-Ag alloy nanourchins, Adv. Mater. 26 (2014) 2431–2439

[S5] W. Niu, Y.A. Chua, W. Zhang, H. Huang, X. Lu. Highly symmetric gold nanostars: crystallographic control and surface-enhanced Raman scattering property, J. Am. Chem. Soc. 137 (2015) 10460−10463.

[S6] I. Al-Ogaidi, H. Goub, A.K.A. Al-kazaz, Z.P. Aguilar, A.K. Melconiana, P. Zheng, N. Wub. A gold@silica core–shell nanoparticle-based surface-enhanced Raman scattering biosensor for label-free glucose detection, Anal. Chim. Acta. 811 (2014) 76–80.

[S7] H. Ilkhan, T. Hughes, J. Li, C.J. Zhong, M. Hepel. Nanostructured SERS-electrochemical biosensors for testing of anticancer drug interactions with DNA, Biosens. Bioelectron. 80 (2016) 257–264.

[S8] S. Su, C. Zhang, L. Yuwen, J. Chao, X. Zuo, X. Liu, C. Song, C. Fan, L. Wang. Creating SERS hot spots on MoS_2 nanosheets with in situ grown gold nanoparticles, ACS Appl. Mater. Interfaces. 6 (2014) 18735–18741.

[S9] M. Ge, C. Wei, M. Xu, C. Fang, Y. Yuan, R. Gua, J. Yao. Ultra-sensitive magnetic immunoassay of HE_4 based on surface enhanced Raman spectroscopy, Anal. Methods. 7 (2015) 6489–6495.

[S10] C. Li, Y. Huang , K. Lai, B.A. Rasco, Y. Fan. Analysis of trace methylene blue in fish muscles using ultra-sensitive surface-enhanced Raman spectroscopy, Food Control 65 (2016) 99–105.

[S11] J. Zhou, F. Zhu, Y. Wang, T. Wang. One-step green synthesis of high uniform SERS substrate based on Au nanoparticles grown on Ge wafer, Chem. Phys. Lett. 627 (2015) 96–100.

[S12] Y. Han, S.L. Lei, J.H. Lu, Y. He, Z.W. Chen, L. Ren, X. Zhou. Potential use of SERS-assisted theranostic strategy based on Fe_3O_4/Au cluster/shell nanocomposites for bio-detection, MRI, and magnetic hyperthermia, Mater. Sci. Eng., C. 64 (2016) 199–207.
